# The Credibility Question: Challenge or Opportunity?

**DOI:** 10.1089/pmr.2020.0010

**Published:** 2020-05-14

**Authors:** Kristin Forner

**Affiliations:** ^1^ Department of Internal Medicine, University of North Carolina, North Carolina, USA.; ^2^ Department of Palliative Medicine, Mission Hospital, Asheville, North Carolina, USA.

The ACGME Common Program Requirements mandate that all surgical training programs provide education in ethical issues specific to the surgical arena. Programs are also responsible both for enhancing the likelihood that residents find meaning in their work and for creating systems that help them manage more challenging cases. In the spring of 2019, experts in the fields of palliative medicine (Kristin Forner, MD), bioethics (Katherine Meacham, PhD), and resiliency (Stephanie Citron, PhD), with the support of the Mountain Area Health Education Center and the Surgery Department at Mission Hospital in Asheville, North Carolina, developed a curriculum to meet these requirements for PGY 1 and PGY 2 general surgery residents.^1^ The motivation of the curriculum was to address the areas within surgical training where all three fields overlap.

Using *Ethical Issues in Surgical Care* (Ferreres, 2017)^2^ as the textual guide, the three course directors created a year-long curriculum consisting of 6 two-hour sessions. Dinner was provided at each session, and the directors facilitated a mixed didactic/discussion format with a brief reflective writing exercise at the conclusion of each session. The six sessions focused on three core bioethical and palliative medicine-related topics: informed consent, adverse events, and end-of-life care.

The first three sessions were attended by all PGY 1 and PGY 2 residents, and were facilitated by the course directors. Surgical faculty members were not allowed to attend, as the course directors assumed that residents would be reluctant to express themselves freely in the presence of surgery faculty. However, after the first three sessions where the course directors noted that resident participation was both minimal and superficial, three surgical faculty members were invited to attend each of the last three sessions. These surgical faculty members' support resulted in dramatically more powerful sessions, in large part because they now held critical, but heretofore missing, credibility. The credibility that came with the surgical faculty involvement (1) allowed for more authentic and meaningful reflections on emotionally charged patient and colleague encounters and technically challenging surgical scenarios and (2) provided accountability when residents drew misguided conclusions or denied the impact of these cases on their individual psyches.

Qualitative outcomes from this curriculum showed that an ethics curriculum can be successfully integrated into a surgical training program. Efficacy is improved, however, if there is involvement of both content experts and surgical faculty. Evidence also showed that the chosen topics provided education in palliative medicine. Given the addition of faculty for the last three sessions, the impact of providing this education was larger and more influential than expected. Surveys of both residents and faculty demonstrated improvement in knowledge of ethical and resiliency issues, as well as the differences between hospice and palliative medicine, and when it may be appropriate to call for a palliative medicine consult.

It is unclear whether such a curriculum will improve resident well-being. By understanding the ethical issues that underlie some of their more challenging cases, and developing a framework with which to discuss or manage them, residents may experience more meaning in their work (Table 1).

**Table 1.** Outline of Hospital Ethics and Resiliency Integrated Training (HEAR-IT) Curriculum

	Topic	Knowledge	Skills	Disposition
Session 1	Informed consent	Shared decision making Obligation to refuse to perform treatments that are harmful	Best case/worst case Offering ONLY medically reasonable options Narrative approach	Empathy Imagination Willingness to accept decisional burden
Session 2				
Session 3	Adverse events	Shame versus guilt Apologies decrease likelihood of lawsuits NC “I'm sorry” statute	Resiliency practices	Courage Compassion for self and others Humility Forgiveness
Session 4				
Session 5	End of life	Surrogacy in NC DNR in OR DCD and brain death	SPIKES protocol Surprise question MOST form	Self awareness Mindful presence Therapeutic silence
Session 6				

DCD, donation after cardiac death; DNR, do not resuscitate; MOST, Medical Order for Scope of Treatment; NC, North Carolina; OR, operating room; SPIKES, Setting, Patient Perspective, Invitation, Knowledge, Emotions, Summary/Strategy.

## Abbreviations Used

DCD: donation after cardiac death
DNR: do not resuscitate
MOST: Medical Order for Scope of Treatment
NC: North Carolina
OR: operating room
PGY: post-graduate year
SPIKES: Setting, Patient Perspective, Invitation, Knowledge, Emotions, Summary/Strategy
